# Mesoporous Silicate Materials in Sensing

**DOI:** 10.3390/s8085202

**Published:** 2008-08-29

**Authors:** Brian J. Melde, Brandy J. Johnson, Paul T. Charles

**Affiliations:** 1 NOVA Research Incorporated, Alexandria, VA 22308, U.S.A.; E-Mail: brian.melde.ctr@nrl.navy.mil; 2 Center for Bio/Molecular Science and Engineering, Naval Research Laboratory, Washington, DC 20375, U.S.A.; E-Mail: paul.charles@nrl.navy.mil

**Keywords:** Mesoporous, silica, organosilica, sensor

## Abstract

Mesoporous silicas, especially those exhibiting ordered pore systems and uniform pore diameters, have shown great potential for sensing applications in recent years. Morphological control grants them versatility in the method of deployment whether as bulk powders, monoliths, thin films, or embedded in coatings. High surface areas and pore sizes greater than 2 nm make them effective as adsorbent coatings for humidity sensors. The pore networks also provide the potential for immobilization of enzymes within the materials. Functionalization of materials by silane grafting or through co-condensation of silicate precursors can be used to provide mesoporous materials with a variety of fluorescent probes as well as surface properties that aid in selective detection of specific analytes. This review will illustrate how mesoporous silicas have been applied to sensing changes in relative humidity, changes in pH, metal cations, toxic industrial compounds, volatile organic compounds, small molecules and ions, nitroenergetic compounds, and biologically relevant molecules.

## 1. Introduction

Mesoporous silicates have been investigated extensively in recent years for use in sensor systems. Some applications of interest include supports for chemical sensing probes, preconcentrators, molecular filters, and hard templates for the preparation of other sensing related materials. According to the International Union of Pure and Applied Chemistry (IUPAC), the prefix meso- refers to a region 2 to 50 nm, macro- is a region > 50 nm, and micro- is a region < 2 nm. The small mesopores limit the kinds of ions and molecules that can be admitted to the interior of the materials. In addition, control over the pore size offers the possibility of molecular sieving or molecular selectivity. Mesoporosity can also endow a material with a high surface area exceeding 1,000 m^2^/g and pore volume greater than 1 cm^3^/g. This greatly expands the potential of the materials for application to adsorption and as a support for immobilized catalytic or sensing moieties.

Sol-gel chemistry is frequently employed in designing these types of silicates [1]. Liquid silicon alkoxide precursors (Si(OR)_4_) are hydrolyzed and condensed to form siloxane bridges, a process that is often described as inorganic polymerization and is represented below:

$$\begin{array}{l} \text{Hydrolysis}:\text{Si}{\left(\text{OR}\right)}_{4}+n{\text{H}}_{2}\text{O}\to {\text{HO}}_{n}‐\text{Si}{\left(\text{OR}\right)}_{4‐n}+n\text{ROH}\\ \text{Condensation}:{\left(\text{RO}\right)}_{3}\text{Si}‐\text{OH}+\text{HO}‐\text{Si}{\left(\text{OR}\right)}_{3}\to {\left(\text{RO}\right)}_{3}\text{Si}‐\text{O}‐\text{Si}{\left(\text{OR}\right)}_{3}+{\text{H}}_{2}\text{O}\\ \text{and}/\text{or}{\;\left(\text{RO}\right)}_{3}\text{Si}‐\text{OR}+\text{HO}‐\text{Si}{\left(\text{OR}\right)}_{3}\to {\left(\text{RO}\right)}_{3}\text{Si}‐\text{O}‐\text{Si}{\left(\text{OR}\right)}_{3}+\text{ROH} \end{array}$$

**Figure 1.**
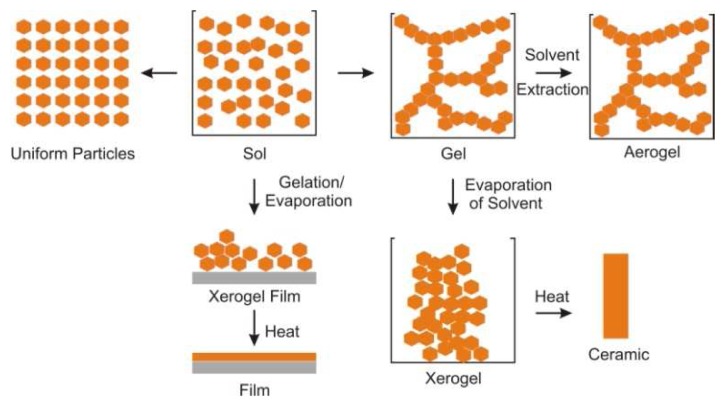
Overview of the sol-gel process illustrating the differences between xerogels and aerogels [1].

The most commonly used precursors are TEOS, tetraethoxysilane, and TMOS, tetramethoxysilane. A colloidal sol of condensed silicate species can eventually interconnect as an immobile three-dimensional network encompassing the space of its reaction container (gel, Figure 1). Drying a gel under ambient conditions or with heat will typically cause shrinkage as solvent leaves the micropores of the silicate network. This type of material is called a xerogel. Alternatively, supercritical drying can be applied to remove solvent yielding a product that is more similar to the size and shape of the original gel. Such aerogels may have low solid volume fractions near 1% and, therefore, very high pore volumes. The use of basic pH and an excess of water can result in particulate precipitation. Gels can also be deposited allowing for the generation of thin films or membranes. The isoelectric point of silica is in the pH range 1-3. This value determines the surface charge of a condensing silicate or material in solution due to protonation and deprotonationation of silanol groups (Si-OH).

**Figure 2.**
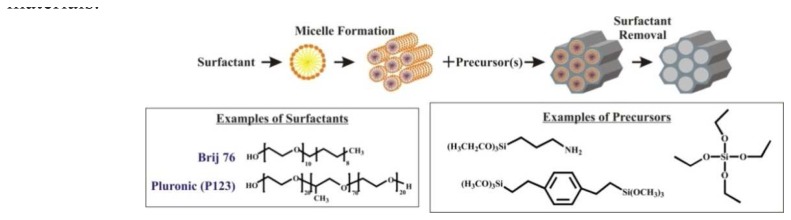
Illustration of the steps involved in the synthesis of surfactant templated silicate materials.

Direct influence over pore size and mesostructure was realized in 1992 with the reports of the M41S materials [2, 3], followed by the introduction of FSM-16 (Figure 2) [4]. Syntheses of M41S materials employs cationic alkylammonium surfactants in amounts above their critical micelle concentrations. These surfactants cooperatively assemble with inorganic precursors to produce an aluminosilicate or silicate matrix. Surfactants are often removed by calcination, or burning, to produce molecular sieves with narrow pore size distributions and highly ordered mesostructures. These types of materials yield reflections in the low angle region of a powder X-ray diffraction pattern. The M41S family includes MCM-41 with two-dimensional hexagonal alignment of mesopore channels, MCM-48 with three-dimensional cubic order, and the layered material MCM-50. FSM-16 is a mesoporous silicate with hexagonal order and is formed by structural rearrangement of layered clay by intercalating surfactant micelles.

Since the first reports of M41S, many different surfactants, precursors and combinations of the two have been studied. Alkylamines have been used to prepare the HMS group of materials [5], the MSU-C series with wormlike mesopore systems were templated with poly(ethylene oxide) surfactants [6], and block copolymers have generated the SBA family of materials[7, 8]. The product designated SBA-15 has hexagonal order like MCM-41 and often features micropores that allow interconnectivity between pore channels. MCM-41 and SBA-15 are the most widely studied and applied templated mesoporous silicas and appear in many of the reports cited in this review. Ordered mesoporous materials have been synthesized using surfactant concentrations above their critical micelle concentrations where “templating” is best described as an inorganic-organic self-assembly process [3, 9]. When greater than 20 wt% surfactant is used, a liquid crystal directs the mesostructure [10, 11]. Liquid crystal templating is used to synthesize mesostructured monoliths with continuous centimeter-scale morphologies. When extraction of templates is used instead of calcinations, organic functional groups can be incorporated into the materials during synthesis.

Silica is an attractive material for many sensing applications because of its stability over a fairly wide range of pH (excluding alkaline), relative inertness in many environments, and transparency in the UV-visible spectrum. Many routes exist for designing hybrid inorganic-organic mesoporous silicates [12]. Silanol groups at the surfaces of mesoporous silicas can be grafted with organosilanes, common examples of which are 3-aminopropyltrimethoxysilane (APTMS), 3-mercaptopropyl-trimethoxysilane (MPTMS), and hexamethyldisilazane (HMDS). In order to achieve a uniform distribution of surface moieties and avoid pore blockage, pores that are large relative to the size of groups to be incorporated are preferred. Hybrid materials called ormosils [13] or ceramers [14] are synthesized directly through co-condensation of silica and organosilica precursors (e.g. Si(OR)_4_ + R′-Si(OR)_3_). This can help to ensure that there is a uniform distribution of groups. Co-condensation can be applied in conjunction with surfactant templating [15-17]. The functional group of an organosilica precursor can associate with the hydrophobic region of the micelles during synthesis, making the group accessible at the mesopore surface upon solvent extraction. A particular class of hybrid material that has been under intense study in recent years is the periodic mesoporous organosilica (PMO) [18-21]. PMOs are usually synthesized using a bridged polysilsesquioxane [22] precursor of the type (RO)_3_Si-R′-Si(OR)_3_ in combination with surfactant templating to produce materials exhibiting mesostructures. These may rival the high degree of order of the pure inorganic products described above.

The synthesis of sol-gel derived mesoporous silicas as particles and gels provides a diversity of applications. Bulk materials may be applied as synthesized (e.g. batch adsorption of an analyte from solution) or as part of a surface coating. Gels can be used to form monolithic materials or thin films on a wide variety of substrates by spin and dip-coating techniques. As will be illustrated in this review, morphological control of mesoporous silicates offers many possibilities for their inclusion in sensing applications.

The scientific literature has become rich in descriptions of the synthesis and functionalization of mesoporous silicates. The last several years have seen the emergence of mesoporous silicate-based sensing applications. Spectrophotometrically active molecular probes can be entrapped in sol-gel glass and applied for heterogeneous detection of analytes in solution or gas. Most studies now take advantage of the high surface areas of mesoporous silicates and silane chemistry to covalently attach molecular probes to the pore walls. This avoids the leaching that may occur from physical encapsulation and mesopores can allow access of analytes to a large number of active sites. For such systems, UV-visible and fluorescence spectrophotometry is often used for quantitative determination of analytes. Another possibility is detection by a visual color change in a material. Pore size can be controlled and surface properties can be altered (e.g. grafting hydrophobic groups) to encourage the entrance of a specific analyte over that of similar species. Mesoporous materials can be used for adsorption and preconcentration of analytes in order to attain detectable concentrations for a particular sensor system. Alternatively, the materials can act as a protective catalytic filter to eliminate an interferent. They can be deposited as or embedded in a specialized coating on an electrode, waveguide, or quartz crystal microbalance to enhance a sensing application. The hydroxylated surface of a mesoporous silicate is wetted by atmospheric moisture, leading to interest in these materials for relative humidity sensing. A mesoporous silica can also be employed as a hard template for the synthesis of a mesoporous material of a composition valuable for sensing.

Some recent reviews have focused on the application of hybrid sol-gel films and monoliths for optical and electrochemical sensing of inorganic species [23]; mesoporous generated the SBA family of materials[24, 25]; zeolites and mesoporous silicates for electrochemical detection [26]; and sol-gels and templated mesoporous materials for fluorescence-based sensing [27, 28]. The versatility of mesoporous silicates has resulted in application to sensors for a wide range of analytes in liquid and vapor phase environments including metal cations, humidity, toxic industrial compounds, volatile organic compounds, nitroenergetics, and biogenic compounds. Concerns that often need to be addressed in the utility of these materials include reversibility and reproducibility, selectivity, response and recovery time, and ease of application.

## 2. Sensors for Relative Humidity

The low conductivity of silica provides a method for monitoring humidity by measuring the change in conductivity. Water molecules interact with hydroxyl sites providing a base for physisorption of water layers as relative humidity increases. For a dry surface at relatively low humidity, conductance occurs through proton “hopping” between the adsorption sites. At higher humidity, water concentrates to form multilayers or condenses to fill a pore. Proton mobility, therefore, becomes more facile and conductivity increases with protons moving from molecule to molecule (Grotthus chain reaction model). A mesopore structure increases the surface area and number of hydroxyl groups available for water adsorption. This is the basis for the investigation of mesoporous silicate thin films as relative humidity sensors. Factors that affect sensor response include the size and accessibility of mesopores, film thickness, number of hydroxyl sites, and organic matter within the pores. Organic matter refers to residual surfactant from a templating process or polymer introduced either during or post synthesis.

As demonstrated by Domansky *et al.*, the number of available hydroxyl and/or silanol sites strongly impacts the potential of a material for application to humidity sensing [29]. Thin films with high surface area (900 m^2^/g) and a disordered pore structure were placed between gold electrodes. It was demonstrated that capping the surface hydroxyl groups through functionalization with hexamethlydisilazane (hydrophobic) resulted in an almost total loss in response to changes in humidity. Ammonia was found to be an interferent and resulted in irreversible damage to these films.

Calcination temperature has been demonstrated to impact the response of a templated thin film to humidity with variations resulting from differences in the number of silanol groups on the surface and the amount of surfactant left in the mesopores. Several studies have compared thin films prepared using templating techniques and calcination between 150 °C and 550 °C [30-33]. It has been shown that lower calcination temperatures resulted in residual surfactant within the pores of the materials. When calcinations below 300°C were used, enhanced sensitivity to low relative humidity (RH) values resulted. Calcinations at higher temperatures provided higher saturation levels. It has been speculated that the residual surfactant in the low calcination temperature materials is the strongest contributing factor to the differences observed. Modification of mesoporous SBA-15 using polypyrrole (PPY) demonstrated this impact as well. When compared to the SBA-15 material, the SBA-15/PPY composite showed a response over a larger range of RH values [34]. Modification of the SBA-15 material using hydrophilic Li^+^ resulted in an impendence change over three orders of magnitude, considerably greater than that of the undoped SBA-15. This change was attributed to the potential for the Li^+^ to dissociate into the adsorbed water increasing the conductivity of the water layers [35, 36]. Complex impedance plots indicated the involvement of protons at low RH values while Li^+^ becomes dominant at higher RH values.

Pore size and accessibility also play a role in the effectiveness of silicate materials used for sensing changes in RH. A comparison of non-templated to templated materials demonstrated an increase in current of several orders of magnitude [37, 38]. Silica aerogels (surface area 866 m^2^/g; average pore size of 20.5 nm; and pore volume 2.83 cm^3^/g.) were compared to a xerogel of lower porosity (surface area 709 m^2^/g, average pore diameter 6.7 nm, and pore volume 1.29 cm^3^/g) [39, 40]. The aerogels were much more sensitive to RH, as measured by impedance and capacitance, than the xerogel. In addition, selection of the electrode materials employed in these types of sensing applications was found to be important. Chromium and gold electrodes provided the most stable current responses while titanium and aluminum showed decreasing responses over extended operation periods [41].

## 3. pH Sensors

A few reports have demonstrated the versatility of mesoporous silicates as active components in pH sensing. These applications typically involve immobilizing an indicating dye on a mesoporous surface or encapsulating it in silicate walls. Incorporation of the dyes on the surface can be accomplished during synthesis or in a post-synthesis grafting process. Fluorescein isothiocyanate (FITC) has been modified with an amino-bearing siloxane to provide a precursor for direct mesoporous material synthesis [42, 43]. The result is a material with FITC on the pore walls which is responsive to pH changes in the range from 3.1 to 11.2 that can be interrogated through laser excited photoluminescence. Grafting of silane-modified 5-methoxy-2-(pyridyl)thiazole (2-MPT) onto SBA-15 has been applied to sensing pH and Cu^2+^ [44]. The material exhibited dual fluorescence emission bands. The first at 420 nm was quenched while the second at 448 nm increased in intensity as pH decreased in the range from 5.7 to 1. Addition of Cu^2+^ at pH 6.0 quenched and blue-shifted fluorescence. A detection limit of 3.2 × 10^-6^ M was obtained. This response was found to be somewhat selective for copper with smaller responses to Fe^3^+ and Hg^2+^. Encapsulation of bromothymol blue provided a material that yielded visual color changes from orange-yellow to royal blue across pH values ranging from 2 to 12. Evanescent wave absorbance spectroscopy (600 nm) was also used for interrogation [45]. Various sulfonephthalein indicators have also been encapsulated in hybrid xerogel films synthesized from mixtures of tetraethoxysilane and vinyltriethoxysilane for application to pH sensing.[46]

**Figure 3.**
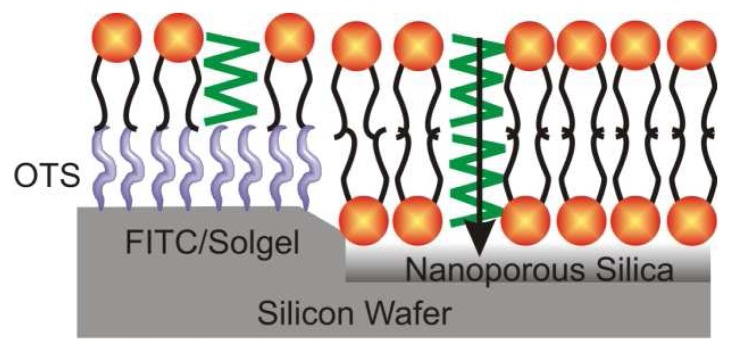
Schematic of a gramicidin A containing lipid membrane organized on FITC-modified mesostructured and mesoporous layers. This system has been applied to sensing changes in the pH of a solution [47].

Yang *et al.* reported an interesting example of combining the functions of biological membranes and nanostructured solids (Figure 3). It involved incorporating fluoresceniosothiocyanate (FITC) by direct synthesis in silica thin films that were patterned into mesostructured and mesoporous regions on a silica wafer [47]. The thin film was coated with a hydrophobic monolayer of *n*-octadecyltrichlorosilane (OTC). A photocalcination procedure was then used to remove all organic components in square patterns. The patterned films were exposed to 1-palmitoyl-2-oleoylphosphatidylcholine (POPC) lipid with gramicidin and a fluorescent lipid. Only a single layer was adsorbed at the hydrophobic OTC monolayers. POPC formed a bilayer in regions with no OTC. In the bilayers, gramicidin could dimerize allowing protons in solution to penetrate into the mesoporous silica. FITC within the mesostructured regions was then used to sense pH changes.

## 4. Metal Cation Sensors

The copper sensitive material described in the previous section is one example among many of mesoporous silicas synthesized for adsorbing and sensing metal cations. Most efforts at cation sensing have relied on either optical or electrochemical sensing methods for detection.

### 4.1. Optical Sensing

Frequently, optical detection of cations relies on dye incorporated into mesoporous materials (Table 1). Interrogation can be accomplished by absorbance or fluorescence measurements. Less commonly, phosphorescence intensities and lifetimes are employed. Some materials may exhibit vivid color changes when exposed to targets providing potential for the design of sensors for interrogation by the naked eye. The mesoporous materials provide high surface area and controlled pore sizes while limiting site accessibility. The limited accessibility can help to shield the dyes from interferents and fouling. Siliated b-diketone was used to modify pore surfaces in ordered mesostructures. The resulting materials were used for the detection of Cu^2+^, Fe^3+^, and U^6+^ as indicated by changes in the UV-visible absorption spectrum of the dye. A detection limit of 1 ppm was reported for U^6+^.[48]

Grafting has been employed to functionalize a material with a porphyrin providing UV-visible detection of Hg^2+^ with a limit of 1.75 × 10^-8^ M (Table 2) [49]. Some interference in the presence of Zn^2+^, Ni^2+^, Pb^2+^, Cd^2+^, and Cu^2+^ was observed. Grafting of 4-(2-pyridylazo)resorcinol into a similar material provided a color change from orange-yellow to purple upon reaction with Cd^2+^ [50]. A detection limit of 1.75 × 10^-8^ M was obtained with some interference from Co^2+^, Ni^2+^, Cu^2+^, and Fe^3+^. Many other cations, anions, and surfactants did not interfere with detection. Grafting of SBA-15 with a silylated calixarene bearing two dansyl fluorophore groups has also been applied to Hg^2+^ detection [51]. Fluorescence emission was quenched upon addition of Hg^2+^ resulting in a limit of detection of 3.3 × 10^-7^ M. Other cations, Na^+^, Pb^2+^, and Cu^2+^, had some impact on intensity, but competed weakly when present with Hg^2+^. Rhodamine modifications have also been used for Hg^2+^ sensing in acetonitrile [52].

Grafting of amino groups onto MCM-48 and MCM-41 has been used for dye immobilization as well [53]. The fluorophores *N*-pyrene-1-yl-succinamic acid and 4-(pyrene-1-yl-carbomoyl)butyric acid have been used for Cu^2+^ sensing in multicomponent mixtures of other metal cations [54]. Here, templated mesoporous silicas were found to be more efficient than modified xerogel supports. Mesoporous materials with a cubic *Fm3m* cage structure were modified with various dyes to provide the potential for sensing of Pb^2+^, Cd^2+^, Sb^3+^, and Hg^2+^ [55, 56]. UV-visible absorption spectrophotometry was used to obtain detection limits of 2.38 × 10^-9^ M (Pb), 13.5 × 10^-9^ M (Cd), 33.7 × 10^-9^ M (Sb), and 6.34 × 10^-9^ M (Hg) with naked eye detection of distinct color changes possible in the nanomolar to micromolar range in appropriate pH ranges.

UV-visible reflectance spectroscopy determined material response times to be under 5 minutes. A similar material with a *Pm3n* cubic cage mesostructure was modified with 4-*n*-dodecyl-6-(2-thiazoylazo)-resorcinol for sensing Pb^2+^ and compared to a functionalized product with a disordered wormhole structure [57]. Although both silicates had three-dimensional mesopore systems, the ordered material had a lower limit of detection of 9 × 10^-9^ M as well as a significantly faster response time and higher diffusion coefficient. Encapsulation of eriochrome cyanine R has been used for evanescent wave based detection of Cu^2+^ with a detection limit of 5 × 10^-5^ M [58].

Gao *et al.* reported a hybrid SBA-15 material used to detect Zn^2+^ and demonstrated how the process of immobilizing a fluorescent probe can actually enhance its activity [59]. SBA-15 was grafted with 3-aminopropyltriethoxysilane so that the amine groups could be used to anchor the Schiff base ligand, 4-chloroaniline-*N*-salicylidene (SC). A previously unobserved emission band was noted in the resulting material upon addition of Zn^2+^. This was believed to be an effect of the π-electron system of the immobilized SC extending through a chlorine moiety on a phenyl ring. A detection limit of 0.2 ng/mL was reported. Selectivity for Zn^2+^ over many other metal cations was observed by its more efficient enhancement of the emission band. An SBA-15 material for Cu^2+^ detection was also generated through grafting [4-(2-hydroxyphenyl)methylene]benzenesulfonamide [60]. Fluorescence was slightly quenched by Cr^3+^, Pb^2+^, and Co^2+^ to the exclusion of many other metal cations. Cu^2+^ quenched the FL significantly to obtain a limit of detection of 0.1 ppm.

Monolithic materials with 3D cubic *Pm3n* and 2D hexagonal *P6mm* geometries were modified with pyrogallol red for Sb^3+^ detection, as was an SBA-15 powder with 2D hexagonal order [61, 62]. Although the materials were all ground to powders before use, those that originally had monolithic morphologies demonstrated faster response times and higher adsorption capacities. It was suggested that this was a function of the larger surface grain sizes of the monolithic materials. Monolithic mesoporous silicates with 3D cubic *Fd3m* geometry were used to sense Bi^3+^ by functionalizing the surfaces with diphenylthiocarbazone [63]. The materials had fast response times of 20-25 s and detection limits ca. 7 × 10^-10^ M.

Functionalized transparent sol-gel monoliths have been used for optical sensing of Cu^2+^ and Cr^6+^ [64-66]. These materials did not rely on dye incorporation but instead used a metal chelating functional group together with spectrophotometric quantification of Cu^2+^ (blue) or Cr^6+^ (yellow). Mercaptopropyltriethoxysilane functionalized SBA-15 has been used to enhance surface plasmon resonance signals on a gold substrate [67, 68]. Hybrid thiol-SBA-15 particles were deposited on Au, and the remainder of the Au surface was functionalized with 1,6-hexanedithiol (HDT). Compared to a Au substrate that was a modified with just a self-assembled monolayer of HDT, the surface with thiol-SBA-15 yielded much larger angle shifts when immersed in 10^-8^-10^-3^ M Pt^2+^ solutions.

### 4.2. Electrochemical Sensing

Mesoporous silicates have been used to modify electrodes for the detection of cations by anodic stripping voltammetry (also referred to as adsorption stripping voltammetry or ASV). This technique involves immersion of the working electrode in a solution of analyte at open circuit for accumulation (or preconcentration). After rinsing, the electrode is placed in a stripping medium, typically containing acid, and a negative potential is applied to reduce the metal cation. The potential is swept toward positive value to reoxidize the metal and regenerate the electrode. The peak current response is measured for sensing purposes. Mesoporous silicas and hybrid organosilicas allow facile access to many active sites, will not swell (as polymers can), and can be formulated to retain their function despite abrasive wear.

While an unmodified silicate material can accumulate metal cations through interactions with anionic silanol groups (Si-O^-^, above isoelectric point) [69, 70], most electrochemical applications rely on surface modifications within the silicate to provide binding affinity. A common modification involves the use of 3-mercaptopropyltrimethoxysilane to provide thiol functional groups on the pore surfaces. Materials of this type have been used for voltammetric sensing of Hg^2+^ and Ag^+^ [71, 72]. Experimentation with materials syntheses confirmed that a regular and highly accessible pore structure strongly influenced the sensitivity of a modified electrode. An optimized thiol-mesoporous silica-functionalized glass carbon electrode (GCE) was able to quantitatively sense Ag^+^ in the concentration range 2 × 10^-7^-1.0 × 10^-5^ M with a detection limit of 6 × 10^-9^ M after a 16 min accumulation period. Thiol functionalization has also been applied through other techniques to the detection of Pb^2+^ and Hg^2+^ [73, 74]. Spin-coating of sols functionalized with thiol, phenyl, and chloropropyl groups has been applied to the detection of Hg^2+^ [75].

Simultaneous detection of Cd^2+^, Cu^2+^, and Pb^2+^ was performed using a carbon paste electrode with an acetamide phosphonic acid (Ac-Phos) monolayer modified silicate material (MCM-41) and desktop square wave voltammetry [76]. Mixing the silicate material with carbon paste provides improved stability under abrasive conditions. Accumulation in a multicomponent solution in the concentration range 10-200 ppb allowed the three cations to be detected together. Higher concentrations resulted in overlap of the peak currents. Similar electrodes were employed in an automated portable ASV sensor where they were inserted in a wall-jet electrochemical cell [77]. This system provided excellent reproducibility with 2.5% relative standard deviation for analysis of Pb^2+^ at low concentrations of 1-25 ppb. The instrument was capable of at least 90 measurements over 5 days; however, there was interference from Cd^2+^ and Cu^2+^. The Ac-Phos and similar salicylamide modified materials were applied using a screen-printing technique for ASV measurements using both handheld and desk-top instruments. Detection of Eu^3+^, Cd^2+^, Pb^2+^, and Cu^2+^ was reported [78, 79].

Functionalized clays, in particular the smectite “Ba” clay from deposits in Central Africa, have been used to modify carbon electrodes for Hg^2+^ sensing [80, 81]. Detection limits of 8.7 × 10^-8^ M and 6.8 × 10^-8^ M were obtained using materials grafted to provide aminopropyl and mercaptopropyl functionality, respectively. The gallery spaces of clays were expanded by intercalation of surfactants and then reacted with mixtures of silane precursors. Functionalized porous clay adhered well to the surface of a GCE by dropping a suspension and drying. A modified electrode obtained a detection limit of 5 × 10^-5^ M Hg^2+^ with linear response from 4× 10^-9^ to 20 × 10^-9^ M.

## 5. Small Molecules and Ions

Detection of oxygen using mesoporous materials is accomplished primarily through optical techniques using an incorporated dye. Oxygen sensing has been accomplished through quenching of the fluorescence of *N*-(3-trimethoxysilylpropyl)-2,7-diazapyrenium bromide incorporated into a highly porous aerogel through direct co-condensation and post-synthesis grafting [91, 92]. It was found that co-condensation provided a more uniform distribution of the dye and more effective interrogation of the resulting photoluminescence. The incorporation of ruthenium complexes into a variety of materials has been applied to detection and quantification of oxygen [82, 92-95]. In the case of the ruthenium complexes, covalent modification techniques were found to yield notably more linear Stern-Volmer plots (I_0_/I vs. oxygen concentration) than techniques that employed entrapment of the dyes [93]. Platinum and palladium metalloporphyrins have been applied to the detection of oxygen through interrogation of fluorescence intensities as well as through phosphorescent lifetime measurements (Table 2) [82-84].

Hydrogen peroxide detection is often accomplished using electrochemical techniques. Mesoporous materials have been used as scaffolds for the immobilization of hemoglobin and myoglobin [96-99]. The silicate materials provide high surface area as well as facilitating electron exchange between the iron of the heme component of these proteins and the electrode. Amine functionalization of the silica provides sites for covalent immobilization of the proteins. Depending on the size of the protein and the pore structure of the silicate material, it is possible to bind the protein either to the surface of the silica or within the pores. In addition to H_2_O_2_ sensing, materials bearing hemoglobin and myoglobin have been applied to the detection of NO_2_. Tyrosinase and horse radish peroxidase have also been immobilized onto MCM-41 to provide a material for application to the detection of phenol.

Mesoporous materials have been applied to the detection of a range of nitrogen-based pollutants including azide, hydrazine, and nitrite [100-110]. Ruthenium oxide modification has been shown to provide a material for the amperometric detection of monomethyl hydrazine with a detection limit of 300 ppb [102, 103]. Copper cryptand moieties have been used for the optical detection of azide with a dynamic range of three orders of magnitude [100]. Silica spheres modified using *p*-dimethylaminobenzaldehyde have been applied to the detection of hydrazine [101]. Selective sensing of nitric oxide has been accomplished in the presence of carbon monoxide through the application of quartz crystal microbalance technology employing a cobalt phthalocyanine modified sol-gel thin film [111]. Electrochemical detection of nitrite has been accomplished using osmium modification of a mesoporous aluminosilicate as well as a metalloporphyrin modified silicate material (Table 2) [85, 86, 112].

An interesting approach was demonstrated by Zhou and coworkers in which a metal-insulator-semiconductor (MIS) design was used for surface photovoltage sensing of NO and NO_2_[105-110]. The MIS structure consisted of a silicon wafer with a silicon dioxide layer and a Si_3_N_4_ layer over that. An ordered mesoporous film was spin-coated as an insulating layer and calcined over the Si_3_N_4_. Al was evaporated on the bottom of the Si and Au was sputtered on top of the mesoporous silica to work as electrodes. An LED light irradiated the Si and induced an AC photocurrent while a DC bias voltage was applied; adsorption of gas changed the dielectric constant of the insulating layer and the measured photocurrent response. Early experiments used pure SBA-15 and SBA-16 films to test the detection of 100 ppm NO in standard air. The greater accessibility of the SBA-16 3D cubic mesostructure yielded greater bias-current shifts in response to targets. MCM-41 thin films incorporating 0.5% tin were found to provide a balance of metal functionality with mesoporosity yielding a detection limit of 100 ppb NO_2_.

Comes *et al.* applied mesoporous silicates for selective sensing of citrate and borate anions by displacement assays [113]. The starting material had a bimodal pore system and was grafted with aminopropyl groups. This material was further modified using either 2-methylthio-2-imidazoline hydroiodide or mannose followed by loading of the materials with dyes. The action of citrate or borate on the materials released dye molecules that could be quantified using UV-visible spectrophotometry. Electrochemical bromate detection has been demonstrated using a material modified with a molybdenum derivative [114]. A xerogel was modified with Pd-doped SnO_2_ nanoparticles to facilitate carbon monoxide sensing [115] while a composite SnO_2_-meosoporous silica has been used to provide H_2_ sensing with significantly higher sensitivity than that of pure tin oxide [116]. Nanocomposites of cobalt oxide and mesoporous silica have been shown to provide a potential material for ozone sensing [117].

## 6. TICs, Pesticides, and Other Targets

Application of mesoporous materials to sensing is not limited to ions and humidity. The high surface area and open pore networks with tunable binding characteristics are ideal for application to the detection of a range of chemical analytes. Detection of ammonia has been accomplished using a mesoporous material modified with Riechardt's betaine dye [118-120]; a material doped with silver nanoparticles [121]; and, through polarimetric interferometry, an unmodified mesoporous material [122]. Benzene detection has been facilitated through interrogation by FTIR of SBA-15 and SBA-16 materials [123]. Other techniques such as measurement of refractive index changes in synthetic mesoporous opals [124] and quartz crystal microbalance using thin films of mesoporous materials have also proven successful [104, 125, 126]. Detection of other common solvents such as toluene and cyclohexane using mesoporous materials has also been reported [90, 127-129]. Sensing of boron trifluoride and boron trichloride, chemicals commonly used in the semiconductor industry, has been accomplished by optical adsorption spectroscopy through the use of dibenzoylmethane modified silica [130].

Modification of mesoporous silica using 7-(*N,N*-dimethylamino)-1-propoxy-3*H*-phenoxazin-3-one resulted in a humidity sensitive material. Further grafting using hexamethyldisilazane resulted in a material with stable fluorescence emission that could be applied to the detection of polar organic vapors such as acetone and methanol [129]. Fluorescence emission of coumarin 485 encapsulated in a mesoporous material, conductance measurements on mesoporous thin films, and quartz crystal microbalance measurements based on mesoporous materials have also been applied to the sensing of alcohols [104, 125, 126, 131, 132]. Thiols and phosphines have been detected using surface plasmon reasonance techniques employing gold nanoparticles supported in a mesoporous thin film [133, 134].

Several reports demonstrate the application of porphyrin-modified mesoporous silicate materials to the detection of nitroenergetic compounds (Table 2). A siloxane-functionalized tetraphenyl porphyrin and metalloporphyrin derivatives of the same structure have been applied to the detection of 2,4,6-trinitrotoluene (TNT), dinitrotoluenes, and nitrobenzene [87-89]. The porphyrin was incorporated into a macrostructured-mesoporous film during the condensation reaction. Detection was accomplished based on quenching of the fluorescence intensity in the presence of target. Another study reported detection of TNT and RDX based on shifts in the fluorescence spectrum of a porphyrin-modified material and compared imprinted and non-imprinted materials [90]. Imprinting of the mesoporous silicate materials is similar to imprinting polymers. A target analog is used during condensation to produce a more favorable binding site on the pore wall. The imprinted material was generated using the bridged polysilsesquioxane precursor 1,4-bis(trimethoxysilylethyl)benzene in order to integrate phenylene functionality throughout the material, thereby, enhancing its binding affinity for TNT. Similar materials lacking the porphyrin modification were used for preconcentration of TNT prior to electrochemical detection [135]. Fluorescence quenching of diazapyrene derivatives within mesoporous materials has also been reported for nitroenergetic sensing [136].

Amine bearing compounds have been detected using a variety of materials. Depending on the type of compound (aminated alkyl chain, aromatic amine, etc) differing approaches provide improved methods. Selective sensing of primary aliphatic amines based on alkyl chain length was accomplished through direct incorporation of methylaniline into a silicate material followed by reaction with 2,6-diphenylpyrilium perchlorate [137]. Increasing the hydrophobicity of the materials through hexamethyldisilizane functionalization was necessary. These materials showed a color change from magenta to yellow for the medium chain amines *n*-heptylamine, *n*-octylamine, and *n*-nonylamine. Shorter and longer chain amines did not produce significant color changes, nor did secondary, tertiary, and aromatic amines. There appeared to be a combination of pore size effects and hydrophobicity that discriminated against more hydrophilic short-chain amines and longer-chain or bulky amines that could block the pore openings. A similar mesoporous silica was functionalized with 1-dicyanomethylene-2-chloro-3-(*N*-methyl-*N*-phenylamino)indene dye moieties and HMDS. This material showed selectivity for *n*-nonylamine and *n*-decylamine, even when in complex mixtures with other amines [138]. Discrimination for relatively small-chain amines and some aromatic amines was demonstrated by anchoring dye moieties on microporous zeolite Beta silicas [139]. Nitrosoamines were detected based on fluorescence quenching using a ZnO modified SBA-15.

Immobilized organophosphoporus hydrolase (OPH) has been applied in a range of schemes for the detection of various organophosphate pesticides and nerve agents. Immobilizing the enzyme within a mesoporous network protects it from bacterial action. In addition, the controlled structure of the silicate material allows for prevention of molecular crowding which can result in a reduction in catalytic activity. A recent study reports on the stability and efficiency of enzymes immobilized within a functionalized mesoporous structure and the optical detection of paraoxon using the material [140]. Glucose oxidase is another enzyme commonly used for electrochemical sensing of glucose. This enzyme has been entrapped in mesoporous materials either singly [141, 142] or in combination with horseradish peroxidase [143] for detection based on amperometric response to the products of the enzyme catalyzed conversion of glucose.

**Figure 4.**
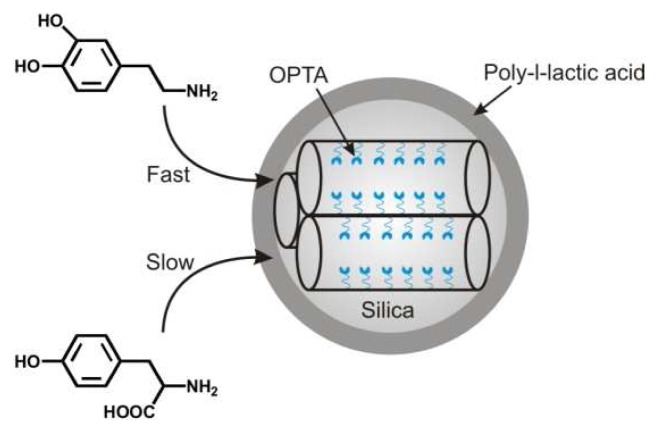
Schematic of poly-l-lactic acid coated mesoporous silica particles. This system has been applied to the selective detection of dopamine in the presence of glutamic acid and tyrosine [145].

Selectivity and specificity were incorporated into thiol-functionalized MCM-41 materials through grafting with propyl, phenyl, or pentafluorophenyl groups [144]. Reaction of the materials with *o*-phthalaldehyde provided *o*-phthalic hemithioacetal (OPTA) moieties. These groups react with dopamine and glucosamine to make highly fluorescent isoindole products. Depending on the functionalization of the materials, varying interaction kinetics and sensitivity to dopamine and glucosamine were observed. The grafted functional groups served to control the diffusion of the targets to the OPTA moieties. In a similar approach, mesoporous silica nanospheres combining “gatekeeping” effect with fluorescent-based detection were designed (Figure 4) [145]. Here, the nanospheres were synthesized with thiol groups, and the outer surfaces of the spheres were grafted with 5,6-epoxyhexyltriethoxysilane. Conversion of epoxy groups to hydroxyl groups provided sites for anchoring poly-l-lactic acid. OPTA moieties were again generated at the thiol-sites within the mesopores. Electrostatic interactions provided selectivity for dopamine over tyrosine and glutamic acid.

Mesoporous silicas combining size selectivity and hydrophobicity have been used for fluorescent sensing of biogenic amines, long-chain carboxylates, and ATP. A pyrilium-methylpyridinium dye was anchored on a mesoporous silicate that was further modified with HMDS [146]. The blue material turned red when placed in a solution containing histamine, putrescine, and cadaverine. No change was observed when exposed to histidine or any of several amino acids. This selectivity was thought to be due to the hydrophobic nature of the material. The limit of detection for histamine was 5 × 10^-4^ M and detection in spiked extracts of the fish *Sparus aurata* was possible. A similar approach using 7-(*N*′-butylureido)-1-methyl-3H-phenoxazin-3-one, a dye that reacts indiscriminately with carboxylates in solution, was applied to the detection of long chain carboxylates such as laurate [147]. Modification of the same material using anthrylmethylamine groups was applied to the detection of ATP [148]. Fluorescence emission from the hybrid materials was stable through a greater pH range before quenching compared to the free probe in solution.

**Figure 5.**
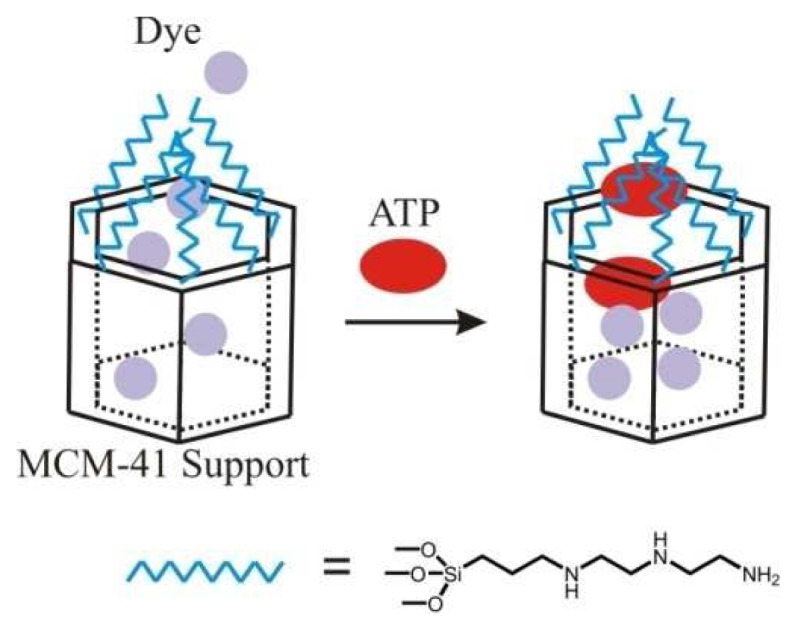
Schematic representation of ATP inhibition of dye release from a gate-like system based on mesoporous silica [149].

Another approach for ATP and ADP recognition involved impregnating a mesoporous silicate with a ruthenium derivative followed by grafting with 3-[2-(2-aminoethylamino)ethylamino]propyltrimethoxysilane (Figure 5) [149]. Most of the polyamine was located at the pore openings. When fully protonated, these groups served as a closed gate to prevent the dye from being released. At neutral and slightly basic pH, the release of dye could be detected. Though anions in general did not block the pore openings, addition of ATP or ADP resulted in blockage of the pores as detected by a lack of observable color in solution. Avidin blocking in biotinylated MCM-41 was used to similar purpose [150]. 2,6-Diaminopyridine immobilized onto mesoporous silica was demonstrated to bind the nucleobases adenosine, cytidine, thymidine, and uracil in water based on fluorescence quenching [151].

## 7. Hard Templates for Sensing Materials

Mesoporous silicates can also be employed as hard templates, or molds, for other compositions. Ordered, well defined mesostructures are particularly suited to these applications. Mesoporous silicates have been used to template carbons, metals, and metal oxides. The silicate framework is usually removed following templating by dissolving with hydrofluoric acid or a strong base. In one example of this technique, Wang *et al.* used 3D cubic mesoporous silica thin films to create Pt nanowire networks by electrodeposition [152]. The Pt networks had a electrochemically active surface area ca. 27 m^2^/g and, when applied as an electrode, exhibited higher current densities for the oxidation of methanol than a non-porous polycrystalline Pt electrode. These materials were applied in a glucose oxidase based glucose sensor and provided approximately 5-fold improvement in sensitivity.

Mesostructured tungsten oxide has been templated by impregnation of 2D hexagonal and 3D cubic mesoporous materials with phosphotungstic acid [153, 154]. In this case materials generated using the 3D cubic silicate were found to provide enhanced sensitivity to NO_2_ likely owing to the more accessible mesostructure. Some materials were also doped with Cu or Cr. XRD showed broad peaks that corresponded to a mixture of monoclinic and triclinic tungsten oxide phases in the nanocrystalline frameworks. A similar technique was applied to the generation of In_2_O_3_ and CaO-In_2_O_3_ materials with SBA-15 [155]. These materials were applied to the detection of CO_2_. Wagner *et al.* synthesized a mesoporous ZnO for sensing CO and NO_2_ by a double hard templating route [156]. First a mesostructured carbon was synthesized by impregnating a mesoporous material with sucrose, pyrolyzing, and removing the silica. The carbon mesostructure was then impregnated with zinc nitrate and heated to convert to ZnO and combust the carbon.

## 8. Conclusions

The majority of the work cited here has been published in the last decade and represents a fairly new and exciting area of interest in the continuously expanding field of mesoporous silicate-based materials. After the reports on the M41S family of mesoporous materials, efforts focused on using various types of surfactants, expansion of mesopore sizes, templating new mesophases, functionalization to prepare hybrid organosilicas, control over macroscale morphology, and application of these concepts to preparing mesoporous versions of other inorganic and hybrid inorganic-organic compositions. Early application developments included modification of mesopore surfaces for heterogeneous catalysis and adsorption of heavy metals and organic solvents.

Basic research into the synthesis and characterization of mesoporous materials will continue, but there appears to be a shift towards proving the worth of these materials in the world outside the laboratory. More work is required to overcome obvious limitations in sensing, but this fact is not surprising considering the relative infancy of this particular area of research. Many of the results discussed here were reported in preliminary or proof-of-concept stages with minimal testing for interference, recoverability, and long term stability. Some studies have begun to investigate these considerations and have found selectivity in the presence of potentially interfering species and retention of sensitivity over several weeks or months. These features should improve with time and the formation of more interdisciplinary collaborations aimed at adaptation of mesoporous silicas to applications involving environmental and biological sensing.

Mesoporous silicates offer high surface area and controlled pore sizes (2-50 nm), both advantages for sensing applications. Some of the results discussed above suggest the influence of the accessibility of the mesopores to be an important issue for detection efficiency. For example, a three-dimensional mesopore system should be more useful for sensing in a thin film than a two-dimensional mesostructure with channels running parallel to the plane of the film. A mesostructure that is highly ordered is less likely to obstruct an analyte with “dead ends” that are more likely in disordered structures. Recent developments in the synthesis of hierarchically structured materials combining improved accessibility with function should benefit sensing applications. Some examples of macrostructured-ordered meosporous silicates include close-packed colloidal crystals of mesoporous silicas [157], inverse opals of mesoporous silica [158], and microphase-separation induced macroporous-mesoporous silica and organosilica monoliths [159, 160]. Macropores can facilitate diffusion enhancing access to the mesopores. This feature should aid in material sensitivity and response and recovery time. Based on reports of periodic mesoporous organosilicas with crystal-like order within the pore walls [161, 162], it may be possible to design macro-meso-microstructured materials with high specific adsorption capacities bearing functional moieties. Research into the application of mesoporous silicas for controlled release of small molecules may also lead to new avenues for sensing, whether related to the release of reporter molecules in response to an analyte or perhaps to the delivery of receptor molecules for interaction with an analyte [24, 25, 163]. Lithographic printing and spin and dip-coating methods provide many opportunities to incorporate mesoporous silicas in sensor systems. The employment of separately functionalized mesoporous silicates in arrays for sensing multiple analytes should be an area of special interest.

## Figures and Tables

**Table 1.** Dyes incorporated for metal cation sensing.

**Dye**	**Cation**	**Detection limit**	**Reference**
Dibenzoylmethane	Uranium (VI)	1 ppm	Nicole *et al.* [48]
Calixarene with two dansyl groups	Mercury (II)	3.3 × 10^-7^ M	Métivier *et al.* [51]
Eriochrome cyanine R	Copper(II)	5 × 10^-5^ M	Miled *et al.* [58]
Meso-tetra(4-sulfonatophenyl)porphine	Mercury(II)	1.75 × 10^-8^ M	Balaji *et al.* [49]
4-(2-pyridylazo)resorcinol	Cadmium(II)	1.75 × 10^-8^ M	Balaji *et al.* [50]
Dithizone α,β,γ,δ-tetrakis(1-methylpyridinium-4-yl)porphine *p*-toluenesulfonate Pyrogallol red meso-tetra(4-sulfonatophenyl)porphine	Lead(II) Cadmium(II) Antimony(III) Mercury(II)	2.38 × 10^-9^ M 1.35 × 10^-8^ M 3.37 × 10^-8^ M 6.34 × 10^-8^ M	Balaji *et al.* [55]
4-chloroaniline-*N*-salicylidene	Zinc(II)	0.2 ng/mL	Gao *et al.* [59]
[4-(2-hydroxyphenyl)methylene]-benzenesulfonamide	Copper(II)	0.1 ppm	Gao *et al.* [60]
2-hydroxybenzaldehyde	Copper(II)	N/A	Zhang *et al.* [53]
*N*-pyrene-1-yl-succinamic acid 4-(pyrene-1-ylcarbamoyl)-butyric acid	Copper(II)	N/A	Kledzik *et al.* [54]
Rhodamine	Mercury(II)^+^	≤ 1.0 × 10^-5^ M	Lee *et al.* [52]
Ethylpyridine with diphenylcarbazide,	Chromium(VI)	10 ppb	Carrington *et al.* [64]
4-*n*-dodecyl-6-(2-thiazoylazo)resorcinol 4-*n*-dodecyl-6-(2-pyridylazo)phenol diphenylcarbazide	Cadmium(II) Lead (II)	0.1 ppb 9 × 10^-9^ M	El-Safty *et al.* [56] El-Safty *et al.* [57]
Pyrogallol red	Antimony(III)	1 × 10^-9^ M	El-Safty *et al.* [61]; Ismail *et al.* [62]
Diphenylthiocarbazone	Bismuth(III)	6.5 × 10^-10^ M	El-Safty *et al.* [63]

**Table 2.** Porphyrin- and metalloporphyrin-based sensing applications.

**Target**	**Porphyrin**	**Reference**
Mercury (II)	*meso*-tetra(4-sulfonatophenyl)porphine	Balaji *et al.* [49, 55]
Oxygen	Pt (II) 2,3,7,8,12,13,17,18-octaethyl porphine Pd (II) 2,3,7,8,12,13,17,18-octaethyl porphine Pt(II) *meso*-tetraphenylporphine Pt (II) *meso* tetra (pentafluorophenyl)porphine	Han *et al.* [82]
Oxygen	Pt(II) *meso*-tetra(4-*N*-pyridyl)porphyrin	Zhang *et al.* [83]
Oxygen	Pt(II) *meso*-tetra(3,5-dihydroxyphenyl)porphyrin Pt(II) *meso*-tetra(3,5-di[(*N*-carbazyl)-*n*-octyloxyphenyl])porphyrin Pt(II) *meso*-tetra(3,5-di[(*N*-carbazyl)-*n*-hexyloxyphenyl])porphyrin Pt(II) *meso*-tetra(3,5-di[(*N*-carbazyl)-*n*-butyloxyphenyl])porphyrin	Huo *et al.* [84]
NO_2_	Co(II) *meso*-tetra(1-methyl-4-pyridyl) porphyrin	Cardoso *et al.* [85, 86]
Nitroenergetic Compounds	*meso*-tetra(4-siloxyphenyl)porphyrin Cd(II) *meso*-tetra(4-siloxyphenyl)porphyrin Zn(II) *meso*-tetra(4-siloxyphenyl)porphyrin	Tao *et al.* [87-89]
2,4,6-Trinitrotoluene RDX *p*-Cresol *p*-Nitrophenol	*meso*-tetra(4-carboxyphenyl)porphyrin	Johnson-White *et al.* [90]
